# Sendai virus-mediated RNA delivery restores fertility to congenital and chemotherapy-induced infertile female mice

**DOI:** 10.1093/pnasnexus/pgae375

**Published:** 2024-09-03

**Authors:** Mito Kanatsu-Shinohara, Hiroko Morimoto, Tianjiao Liu, Masaru Tamura, Takashi Shinohara

**Affiliations:** Department of Molecular Genetics, Graduate School of Medicine, Kyoto University, Yoshida Konoe, Sakyo-ku, Kyoto 606-8501, Japan; AMED-CREST, AMED, 1-7-1 Otemachi, Chiyodaku, Tokyo 100-0004, Japan; Department of Molecular Genetics, Graduate School of Medicine, Kyoto University, Yoshida Konoe, Sakyo-ku, Kyoto 606-8501, Japan; Department of Molecular Genetics, Graduate School of Medicine, Kyoto University, Yoshida Konoe, Sakyo-ku, Kyoto 606-8501, Japan; Technology and Development Team for Mouse Phenotype Analysis Division, RIKEN BioResource Research Center, 3-1-1 Koyadai, Tsukuba, Ibaraki 305-0074, Japan; Department of Molecular Genetics, Graduate School of Medicine, Kyoto University, Yoshida Konoe, Sakyo-ku, Kyoto 606-8501, Japan

**Keywords:** blood-follicle barrier, infertility, Kitl, ovulation, Sendai virus

## Abstract

Current infertility treatment strategies focus on mature gametes, leaving a significant proportion of cases with gamete progenitors that stopped complete differentiation. On the other hand, recent advancements in next-generation sequencing have identified many candidate genes that may promote maturation of germ cells. Although gene therapy has shown success in mice, concerns about the integration of DNA vectors into oocytes hinder clinical applications. Here, we present the restoration of fertility in female mice through Sendai virus (SeV)-mediated RNA delivery. Ovaries lacking *Kitl* expression exhibit only primordial follicles due to impaired signaling to oocytes expressing the KIT tyrosine kinase. Despite SeVs being immunogenic and larger than the blood-follicle barrier, the administration of *Kitl*-expressing SeVs reinitiated oogenesis in genetically infertile mice that have only primordial follicles, resulting in the birth of normal offspring through natural mating. This virus also effectively addressed iatrogenic infertility induced by busulfan, a widely used cancer chemotherapy agent. Offspring born through SeV administration and natural mating displayed normal genomic imprinting patterns and fertility. Since SeVs pose no genotoxicity risk, the successful restoration of fertility by SeVs represents a promising approach for treating congenital infertility with somatic cell defects and protecting fertility of cancer patients who may become infertile due to loss of oocytes during cancer therapy.

**Significance Statement**

Infertility rates are rising globally. Existing infertility treatments are restricted to patients with mature gametes, leaving many with premature ovarian failure remain infertile, prompting exploration of treatments like gene therapy. Concerns about virus vector integration into the germline have limited its adoption. Here, we demonstrate Sendai viruses (SeVs) effectively treating both congenital and iatrogenic infertility in mice. SeVs lack a DNA phase, reducing genotoxicity risk. Despite SeVs' immunogenicity in other organs, the ovaries exhibit unique immunotolerance, enabling successful offspring production through natural mating. SeVs also rescued busulfan-induced infertility by supplying a cytokine that promotes oocyte survival. With no genotoxicity, SeVs offer a promising approach for treating congenital infertility and preserving fertility during cancer therapy by promoting oocyte survival.

## Introduction

Oogenesis is a protracted and intricated process regulated by a multitude of intra- and extra-ovarian factors (1, 2). Consequently, mutations in genes involved in this regulation result in infertility. The growth and functional maturation of oocytes require signals not only from surrounding somatic cells but also from the hypothalamus–pituitary–ovarian axis via hormonal stimulation. There are three phases of oocyte development both in vitro and in vivo: in vitro differentiation “IVD” which is not gonadotropin sensitive, in vitro gonadotropin sensitivity “IVG”, which is the phase of gonadotropin stimulation to prepare for meiotic competence, and in vitro maturation to metaphase II (3). Bidirectional communication between oocytes and surrounding granulosa cells is critical for the maturation of both oocytes and for granulosa cells. Granulosa cells supply necessary nutrients and metabolites to oocytes through gap junctions (4). Although next-generation sequencing technologies have identified many candidate genes responsible for female infertility (5, 6), it is not easy to define candidate genes in humans and there are currently few methods for their treatment. In addition to congenital infertility, recent developments in cancer treatment have increased the number of cancer survivors who become infertile (7, 8). This is particularly true for prepubertal patients, where approximately half of the patients become infertile despite an 80–90% of survival rate (9, 10). Although assisted reproduction techniques, such as in vitro fertilization (IVF) or intracytoplasmic sperm injection (ICSI), are widely used for infertility treatment, these techniques can only be applied when mature gametes are available. Moreover, studies in mice revealed potential transgenerational defects associated with these techniques, raising concerns about human offspring (11). Therefore, developing new techniques to rescue infertile female patients is an urgent problem.

One potential therapeutic approach for infertility is gene therapy. Recent clinical trials of gene therapy for somatic cells have demonstrated remarkable therapeutics benefits and an excellent safety record (12). In particular, lentiviruses (LVs) and adeno-associated viruses (AAVs) are extensively used for hematopoietic stem cell, liver, T cell, and retinal gene therapies in humans. However, few attempts to treat infertility have been reported. To date, LVs, adenoviruses (AVs), and AAVs have been used for treating congenitally infertile male and female mice, resulting in the birth of normal offspring with appropriate genomic imprinting patterns (13–18). Mice carrying single gene mutations that cause somatic cell dysfunction were successfully cured in these studies. Importantly, none of the offspring produced by gene therapy contained exogenous transgenes, demonstrating the safety of gene therapy in infertility treatment. However, several studies on somatic cells revealed that currently used gene therapy vectors can integrate into the host genomes. For example, detailed analyses on long-term studies of AAV-based therapies for hemophilia A in dogs revealed numerous integrations of vectors with partial deletions or rearrangements (19). In mouse experiments with gene therapy, at least part of the risk appears to be due to the integration of AAV vectors at a particular locus of the mouse genome (20). Therefore, the risk of genotoxicity in humans is not negligible, despite successful mouse experiments for infertility treatment.

Sendai viruses (SeVs) are murine parainfluenza viruses of type 1, belonging to the family Parmyxoviridae (21, 22). SeVs are characterized by a single-strand RNA genome, and a linear and nonsegmented negative-strand RNA of ∼15.4 kb. They have been utilized in gene therapy for several reasons. First, SeVs undergo cytoplasmic replication cycles, eliminating the risk of integration into genomic DNA as there is no DNA phase during their life cycles. Second, their infection efficiency is independent of the cell cycle of the target cells. Third, SeVs have no association with human diseases. Fourth, they infect a broad range of host cells by utilizing the sialic acid-conjugated glycoproteins and glycolipids as receptors for binding. Finally, paramyxoviruses, including SeVs, exhibit low genetic recombination or instability, and no homologous or heterologous recombination has ever been reported for SeVs. Due to these properties, SeVs have been considered a safe strategy to produce induced pluripotent stem cells for regenerative medicine (23).

On the other hand, SeVs have several disadvantages. One of them is their strong induction of immune response. The innate immunity against SeVs is particularly severe. A previous study showed that cultured cells infected with SeVs were promptly rejected not only in wild-type mice but also in nude mice (24). This challenge could be overcome by using NOG mice, which lack not only T cells but also B cells and NK cells. RIG1 has been identified as particularly crucial for sensing RNA viruses (25). Another significant limitation arises from platelet agglutination induced by hemagglutinin–neuraminidase (HN) protein. Although HN protein on SeVs is essential for cell fusion, it causes agglutination of red blood cells (26, 27), thereby restricting intravenous administration. Hemagglutination occurs when the HN protein binds to sialic aid on the surface of red blood cells, while both F and HN proteins induce hemolysis. Lastly, the cargo size of SeVs is similar to that of AAVs but much smaller than those of LVs or AVs, limiting the range of target diseases.

In the present study, we employed SeVs for infertility treatment in mice. Although SeV infection triggers an immune response, we tested whether SeV might facilitate the successful rescue of infertility. Because SeVs are injected directly into the ovaries, the risk of hemagglutination-induced damage is expected to be much lower than injection into blood vessels. We microinjected SeVs into congenitally infertile *Kitl^Sl-t^/Kitl^Sl-t^* mice (28), which lack *Kitl* expression completely in the ovaries (15). KIT is expressed in granulosa cells, while its tyrosine kinase receptor KIT is expressed in oocytes and theca cells (29, 30). Due to the complete absence of *Kitl* expression in ovaries, *Kitl^Sl-t^/Kitl^Sl-t^* mice only contain primordial follicles, and no secondary follicles are found in mature ovaries (28). Therefore, they can serve as a model for premature ovarian insufficiency in humans, estimated to affect 0.3 to 1.1% of women of reproductive age (31). The same virus vector also rescued chemotherapy-induced iatrogenic infertility in wild-type mice, suggesting that SeVs are applicable for fertility protection against cancer treatment (7). Our successful production of offspring from infertile mouse models opens new possibilities for applying SeVs to the treatment of female infertility.

## Results

### Introduction of SeVs into wild-type ovaries

**Fig. 1.**
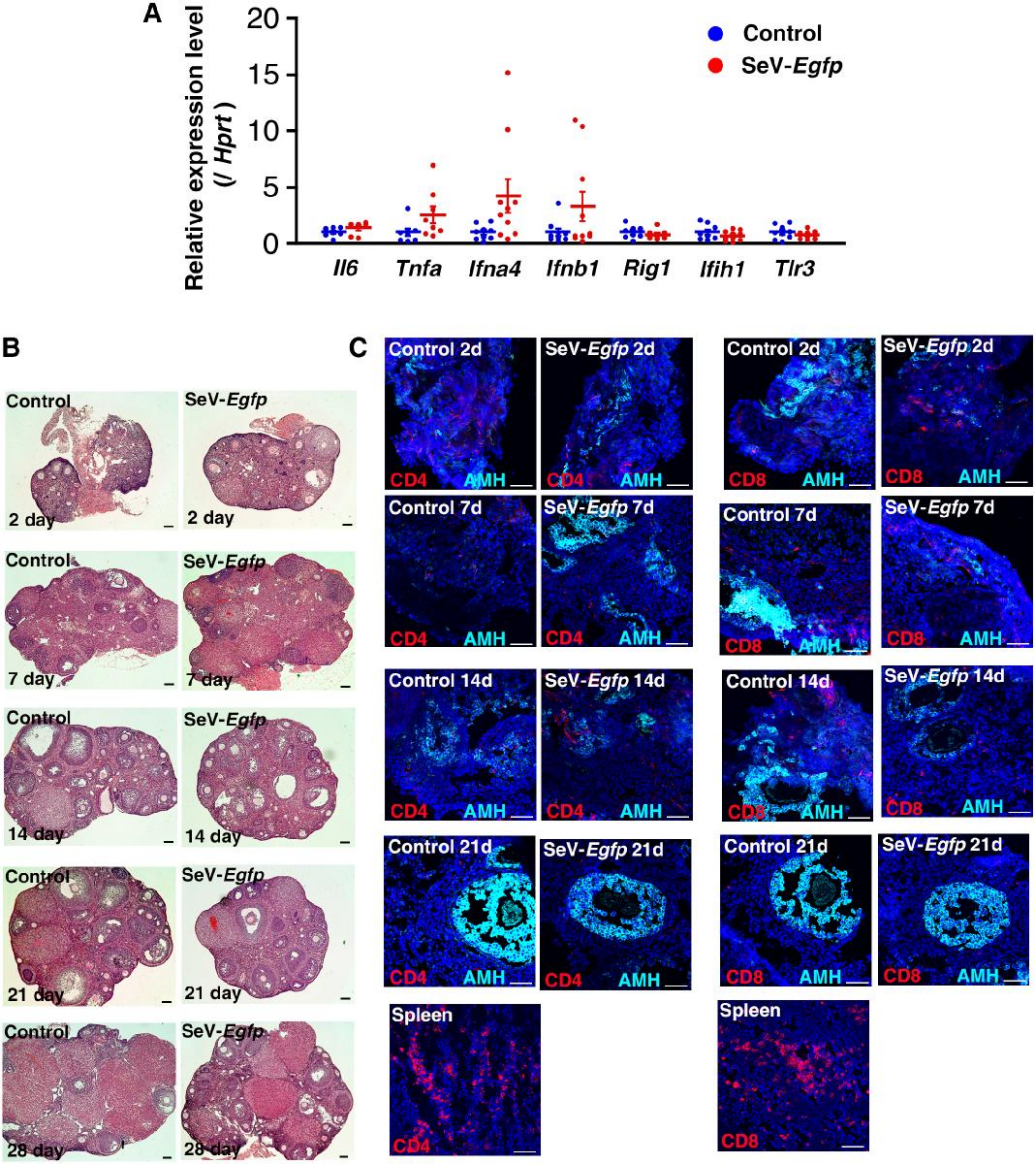
Introduction of SeVs into wild-type ovaries. A) Real-time PCR analysis of ovaries (*n* = 7 for *Il6*, *n* = 8 for *Tnfa*, *n* = 9 for control of *Ifna4*, *Ifnb1*, and *Tlr3* and *n* = 8 for control of *Rig1*, and *Ifih1*, *n* = 10 for others). Data are represented as mean ± SEM. B) Histological analysis of ovaries. C) Immunostaining with lymphocyte markers. Bar = 100 μm (B), 50 μm (C). Stain: hematoxylin and eosin (B), Hoechst33342 (C).

One challenge in utilizing SeVs for infertility treatment is their high immunogenicity (24). To assess the impact of SeV introduction into ovaries, we microinjected SeVs expressing *Egfp* (SeV-*Egfp*) into the ovaries of wild-type C57BL/6 (B6) mice. Ovaries were collected 1 week after microinjection, and real-time PCR was conducted to identify changes in gene expression related to innate immunity. Real-time PCR analysis revealed that SeV administration did not induce typical molecules associated with inflammation, such as *Il6*, *Tnfa*, *Ifna4*, *Ifnb1*, *Rig1*, *Ifih1*, and *Tlr3* (Fig. 1A).

To further confirm the potential inflammation, ovaries were collected at various time points (2, 7, 14, 21, and 28 days), and the development of inflammatory reactions were examined. Histological analysis of ovaries did not reveal apparent inflammation and fibrosis in any of the collected samples (Fig. 1B). Oogenesis continued normally in ovaries injected with SeVs. To confirm the lack of inflammation, immunostaining was performed and the invasion of inflammatory cells was evaluated. Consistent with the histological analysis results, immunostaining with anti-CD4 or anti-CD8 antibodies did not indicate apparent infiltration of lymphocytes (Fig. 1C). While CD4 or CD8 lymphocytes were occasionally observed, their numbers remained unchanged during the experimental period and were comparable to those in control ovaries. In contrast, immunostaining of the spleen showed strong positive signals. These findings suggest that the ovaries are a unique organ, preventing apparent inflammatory reactions despite SeV infection.

### Defective blood-follicle barrier in Kitl^Sl-t^/Kitl^Sl-t^ mice

While SeVs exhibited limited inflammatory reactions in ovaries, their size is larger than the molecular sieve of the blood-follicle barrier (BFB) (32, 33). Therefore, it was possible that SeVs might not penetrate the BFB, which prevents the penetration of exogenous molecules into ovarian follicles (34). We performed double immunostaining to confirm the infected cell types using SeV-*Egfp* and ovarian cell markers. Although EGFP signals were found in HSD3B-expressing theca cells, no apparent signals were detected in cells within ovarian follicles (Figure S1). Neither AMH-expressing granulosa cells nor MVH-expressing oocytes exhibited EGFP fluorescence. These results suggested that SeVs cannot penetrate the BFB.

Even though these findings indicate limited application of SeVs in intrafollicular cell–cell interaction, we hypothesized that the BFB may be defective in infertile animals, as several studies suggested a defective blood-testis barrier in infertile male mice (35). To test this possibility, we examined the BFB in *Kitl^Sl-t^/Kitl^Sl-t^* mice, which completely lack the expression of *Kitl* expression in ovaries (15). The KITL–KIT interaction is indispensable for successful oogenesis. It is considered that KITL expressed on granulosa cells binds to the KIT tyrosine kinase receptor on oocytes and triggers a signaling cascade to support survival and differentiation of oocytes (36). Consequently, *Kitl^Sl-t^/Kitl^Sl-t^* mice lack oogenesis but contain a significant number of primordial follicles with one layer of granulosa cells (15). These mice are congenitally infertile and serve as a model for premature ovarian failure.

**Fig. 2.**
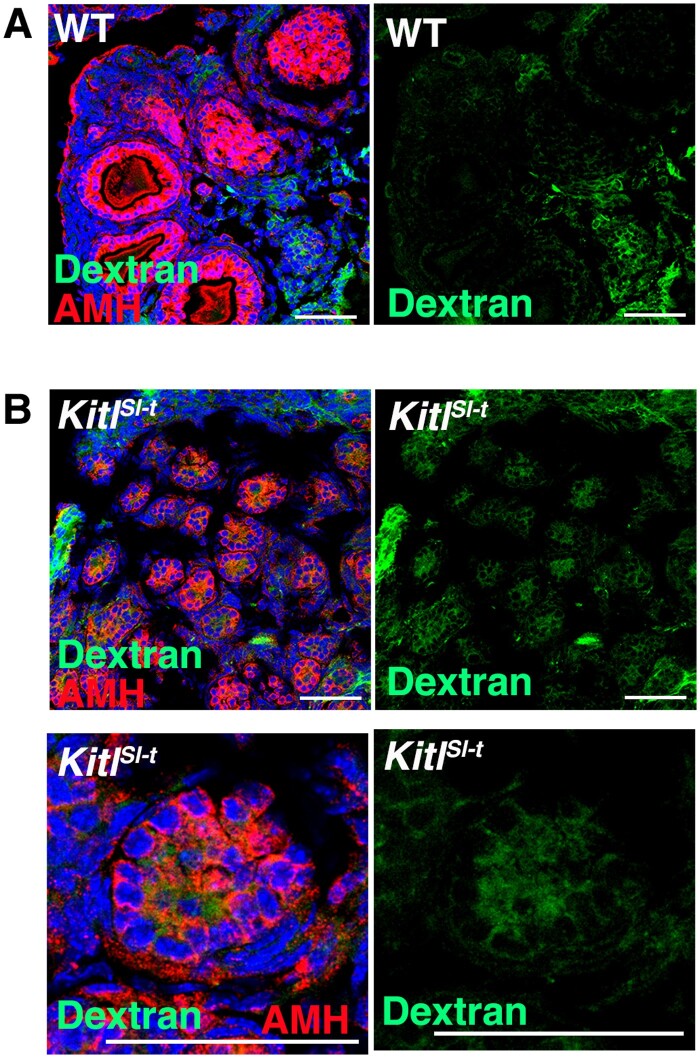
Immunostaining of ovaries after microinjection of FITC-dextran. A) Wild-type ovary. B) *Kitl^Sl-t^/Kitl^Sl-t^* ovary. Bar = 50 μm (A, B). Stain: Hoechst33342 (A, B).

We prepared fluorescence isothiocyanate (FITC)-dextran, which has a molecular size of 10 K, which cannot penetrate the BFB. We microinjected FITC-dextran into *Kitl^Sl-t^/Kitl^Sl-t^* and wild-type mice, and their ovaries were recovered 30 min after microinjection. Analysis of the ovaries showed FITC signals exclusively in interstitial regions of wild-type ovaries (Fig. 2A). However, when the same solution was microinjected into *Kitl^Sl-t^/Kitl^Sl-t^* ovaries, FITC signals were found within the ovarian follicles (Fig. 2B).

To confirm the defective BFB, we introduced SeVs that expressed *Egfp.* As expected, EGFP signals were found in AMH-expressing granulosa cells, HSD3B-expressing theca cells, and MVH-expressing oocytes (Figure S2A–C). The proportion of primordial follicles that expressed EGFP was 61.0 ± 14.2 and 34.8 ± 9.0% (*n* = 3 ovaries) of AMH^+^ granulosa cells showed fluorescence. We also injected SeV-*Egfp* into *Lhcgr* knockout (KO) mice and WBB6F1-W/W^v^ (W) mice. The former have a defective hypothalamus–pituitary–gonadal (HPG) axis and lack preovulatory follicles, while the latter lack KIT and have few germ cells (35, 37). In both models, we observed EGFP fluorescence not only in theca cells but also in granulosa cells. However, while EGFP signal was confirmed in oocytes of *Lhcgr* KO mice, oocytes in W mice were too few and apparent signals were not found in residual oocytes, which could be due to poor penetration of SeVs. These results suggested that mice with impaired oogenesis have defective BFB, raising the possibility of transducing granulosa cells with SeVs in *Kitl^Sl-t^/Kitl^Sl-t^* ovaries.

### Introduction of Kitl genes into wild-type ovaries

While these results suggested the feasibility of SeVs to penetrate the BFB, several potential problems may arise when *Kitl* is overexpressed in ovaries. For instance, long-term cytokine overexpression might lead to the development of tumors (38). As *Kitl* is crucial in organizing the theca layer around the developing follicle (39), SeV expression could potentially disturb oogenesis. To investigate the impact of *Kitl* overexpression on fertility in wild-type mice, we injected *Kitl*-expressing SeVs (SeV-*Kitl*) and used SeV-*Egfp* as a control. After virus injection, mice were housed with wild-type males 2 weeks post-microinjection, and the animals were maintained for 3 months to assess the efficiency of offspring production.

**Fig. 3.**
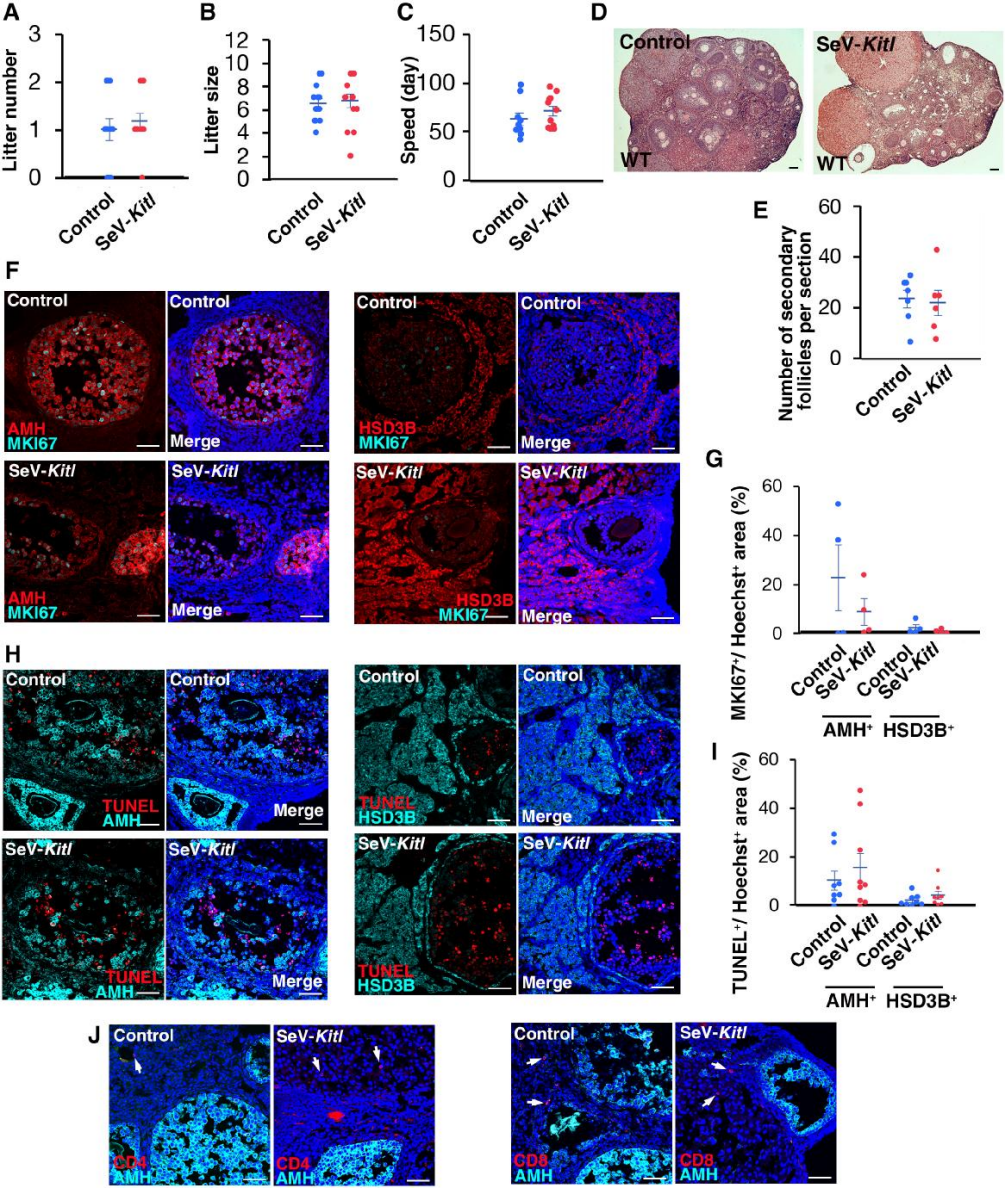
Functional analysis of SeV-*Kitl* injection into wild-type mice. A) Litter number (*n* = 13 for control, *n* = 12 for SeV-*Kitl*). Data are represented as mean ± SEM. B) Number of offspring per litter (*n* = 13 for control, *n* = 14 for SeV-*Kitl*). Data are represented as mean ± SEM. C) Speed of offspring production (*n* = 9 for control, *n* = 11 for SeV-*Kitl)*. Data are represented as mean ± SEM. D) Histological analysis of ovaries. E) Number of secondary follicles per ovary (*n* = 7 for control; *n* = 6 for SeV-*Kitl*). Data are represented as mean ± SEM. F) Immunostaining of MKI67. G) Number of MKI67^+^ cells per follicle (*n* = 4). Data are represented as mean ± SEM. H) TUNEL staining. I) Quantification of apoptotic cells (*n* = 8). Data are represented as mean ± SEM. J) Immunostaining with lymphocyte markers (arrows). Bar = 100 μm (D), and 50 μm (F, H, J). Stain: hematoxylin and eosin (D), Hoechst33342 (F, H, J).

In total, 11 of 12 mice were fertile after SeV-*Kitl* injection, while 9 out of 13 control mice produced offspring. Over the 3 months, 14 litters were born from 11 females transduced with SeV-*Kitl*, whereas a total of 13 litters were born from 9 control females transduced with SeV-*Egfp* during the same period (Fig. 3A). For Sev-*Kitl,* the litter size ranged from 2 to 9 with an average of 6.7, and for SeV-*Egfp*, the litter size ranged from 4 to 9 with an average of 6.5. There were no apparent differences in litter size between the 2 types of mice (Fig. 3B). In terms of the speed of offspring production, however, one of the 12 female injected with SeV-*Kitl* produced offspring as early as 50 days after microinjection. On the other hand, the first offspring from 13 SeV-*Egfp* mice were born after 40 days. The average time taken for the first litter production was 67.1 and 59.4 days for SeV-*Kitl* and SeV-*Egfp*, respectively (Fig. 3C). The difference was not statistically significant. Because widespread *Kitl* overexpression may trigger differentiation, it was possible that *Kitl* induction can lead to the depletion of the oocyte pool and infertility. However, 3 out of 12 Sev-*Kitl*-injected mice were able to sire 2 litters within 3 months. Similarly, 4 out of 13 female mice injected with the control vector produced 2 litters. These differences were not statistically significant.

Three months after microinjection, all recipient mice were sacrificed for histological analysis of ovaries. The size of the ovaries did not change significantly between the two samples. Histological analysis showed normal appearing oogenesis in all the mice injected with SeV-*Kitl* (Fig. 3D). Consistent with the fertility analysis, the number of secondary oocytes did not show apparent differences (Fig. 3E). Immunostaining of ovaries did not reveal apparent changes in the number of MKI67^+^ granulosa cells between SeV-*Kitl-* and SeV-*Egfp* ovaries (Fig. 3F and G). We also performed TdT-mediated dUTP nick end labeling (TUNEL) staining to check whether excessive *Kitl* expression might have reduced apoptosis. However, no apparent differences were found between the two samples (Fig. 3H and I). Although CD4 or CD8 lymphocytes were occasionally detected, their numbers did not change significantly (Fig. 3J).

To check the potential infection of oocytes by SeVs, genomic DNA was collected from the offspring, PCR was performed to investigate the potential integration of SeV-*Egfp-*specific primers (Figure S3). However, none of the offspring showed evidence of integration into the genome of the offspring. These results indicated that SeV-*Kitl* expression allowed the complete oogenesis without the risk of transgene integration into the germline cells.

### Induction of oogenesis in Kitl^Sl-t^/Kitl^Sl-t^ mice

**Fig. 4.**
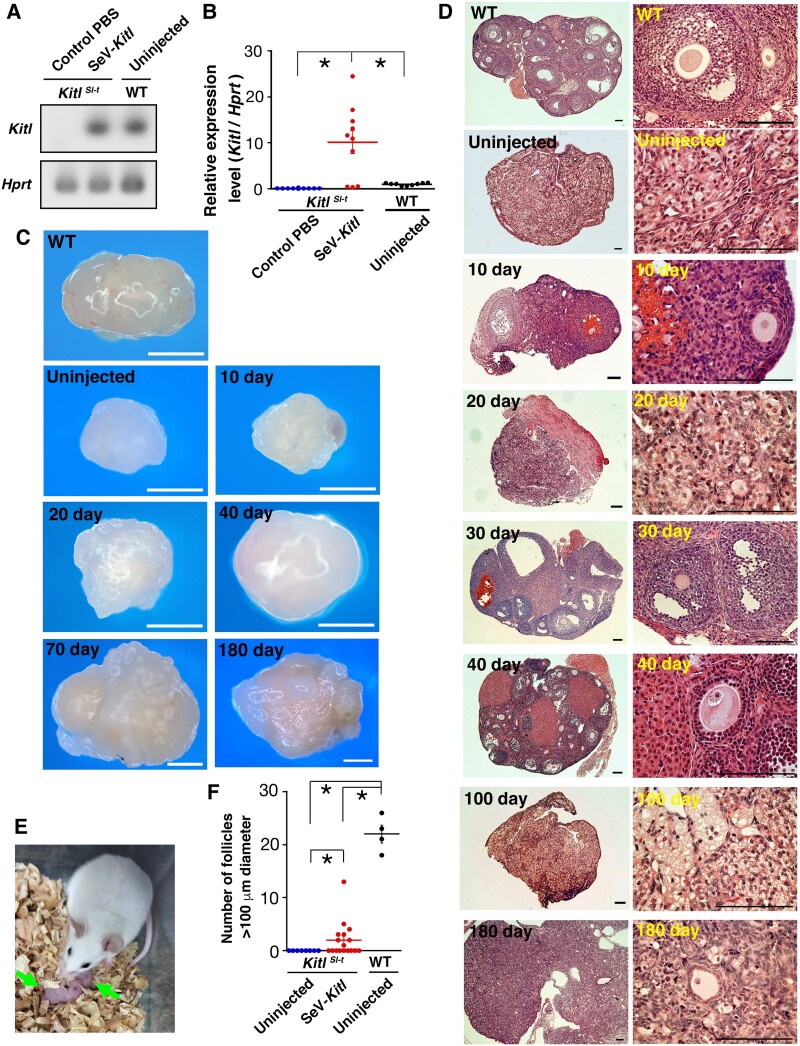
Restoration of fertility in *Kitl^Sl-t^/Kitl^Sl-t^* mice. A, B) RT-PCR (A) and Real-time PCR (B; *n* = 9 for WT uninjected, *n* = 10 for others) analyses of *Kitl* expression. The relative expression levels were compared with that in WT mice. Data are represented as mean ± SEM. C) Appearance of ovaries after SeV-*Kitl* injection. D) Histological analysis of ovaries. E) Offspring (arrows) born after mating with *Kitl^Sl-t^/Kitl^Sl-t^* mutant males. F) Analysis of follicle development 6 months after SeV-*Kitl* injection (*n* = 8 for, *Kitl^Sl-t^* uninjected; *n* = 17 for SeV-*Kitl*; *n* = 4 for WT uninjected). Bar = 1 mm (C), 100 μm (D). Stain: hematoxylin and eosin (D).

To test whether SeV-*Kitl* can restore congenital infertility, we microinjected SeV-*Kitl* into mature *Kitl^Sl-t^/Kitl^Sl-t^* mice. Both ovaries received SeV-*Kitl* injection. Ovaries were collected on the following day after injection, and RT-PCR and real-time PCR were performed to confirm the expression of *Kitl*. Our analysis revealed a 10.1-fold increase in *Kitl* expression compared with wild-type control (Fig. 4A and B). To assess the impact of *Kitl* overexpression, mice were sacrificed at 1, 3, and 12 weeks after SeV injection. The size of the ovaries increased following virus injection (Fig. 4C). Histological analysis of the ovaries indicated follicle development upon SeV-*Kitl* microinjection (Fig. 4D). Subsequently, we mated the remaining animals for natural fertility. *Kitl^Sl-t^/Kitl^Sl-t^* mutant females were housed with *Kitl^Sl-t^/Kitl^Sl-t^* males, as the *Kitl^Sl-t^/Kitl^Sl-t^* males are fertile.

In total, 5 of 22 (22.7%) mice across 3 separate experiments sired progeny as early as 50 days after SeV injection (Fig. 4E). The first litter was obtained on average of 68.1 days after virus injection. One female sired 2 litters, but no offspring were born after 99 days. Thirteen offspring were born in total, consisting of four males and nine females. The average litter size was 2.2 per litter. No apparent morphological abnormalities were observed in these offspring, and they grew up normally, exhibiting a white coat color (Figure S4), consistent with *Kitl^Sl-t^/Kitl^Sl-t^* mutant mice lacking melanocytes, a trait dependent on KITL–KIT signaling.

Subsequently, we examined hormonal levels after SeV-*Kitl* injection (Figure S5A–C). Blood samples were collected at different time points, and enzyme-linked immunosorbent assay (ELISA) was performed to measure the levels of follicle stimulating hormone (FSH), luteinizing hormone (LH), and estrogen. However, none of the hormones showed significant changes. Despite apparently abnormal hormonal levels, ovulation and pregnancy occurred, as mature follicle ovulation is typically induced by a burst of LH secretion in normal females.

All *Kitl^Sl-t^/Kitl^Sl-t^* mice were sacrificed 6 months after SeV microinjection. Although the size of *Kitl^Sl-t^/Kitl^Sl-t^* ovaries was smaller than those of wild-type mice, they were larger than ovaries of *Kitl^Sl-t^/Kitl^Sl-t^* mice that did not receive virus injection (Figure S6). Histological sections of ovaries from SeV-*Kitl* injected mice showed a normal appearance with varying levels of oogenesis, whereas no follicles developed in ovaries that did not receive the virus (Fig. 4D). There was a significant increase in the number of follicles with a diameter exceeding 100 μm (Fig. 4F). However, the number of follicles was significantly lower compared to wild-type ovaries, which may explain the relatively small litter size after SeV microinjection. Nevertheless, these results suggest that SeV-*Kitl* microinjection can rescue and maintain long-term oogenesis.

### Restoration of infertility in busulfan-treated mice

The results presented in the preceding sections highlight the utility of SeV in treating congenital infertility. Building on this observation, we investigated the potential of using the same virus for busulfan-induced infertility. Busulfan, a chemical reagent widely employed in cancer treatment, can also impair fertility. Wild-type B6 mice were administered busulfan, resulting in a significant loss of ovarian follicles, as indicated in previous studies (40). Given that KITL provides survival signals, we hypothesized that overexpression of KITL in busulfan-treated mice might protect fertility by enhancing survival signals. SeV-*Kitl* or SeV-*Egfp* was microinjected into 10 mice, which were then subjected to busulfan treatment. Subsequently, the fertility of the injected mice was assessed by mating the female mice with wild-type males for 3 months.

**Fig. 5.**
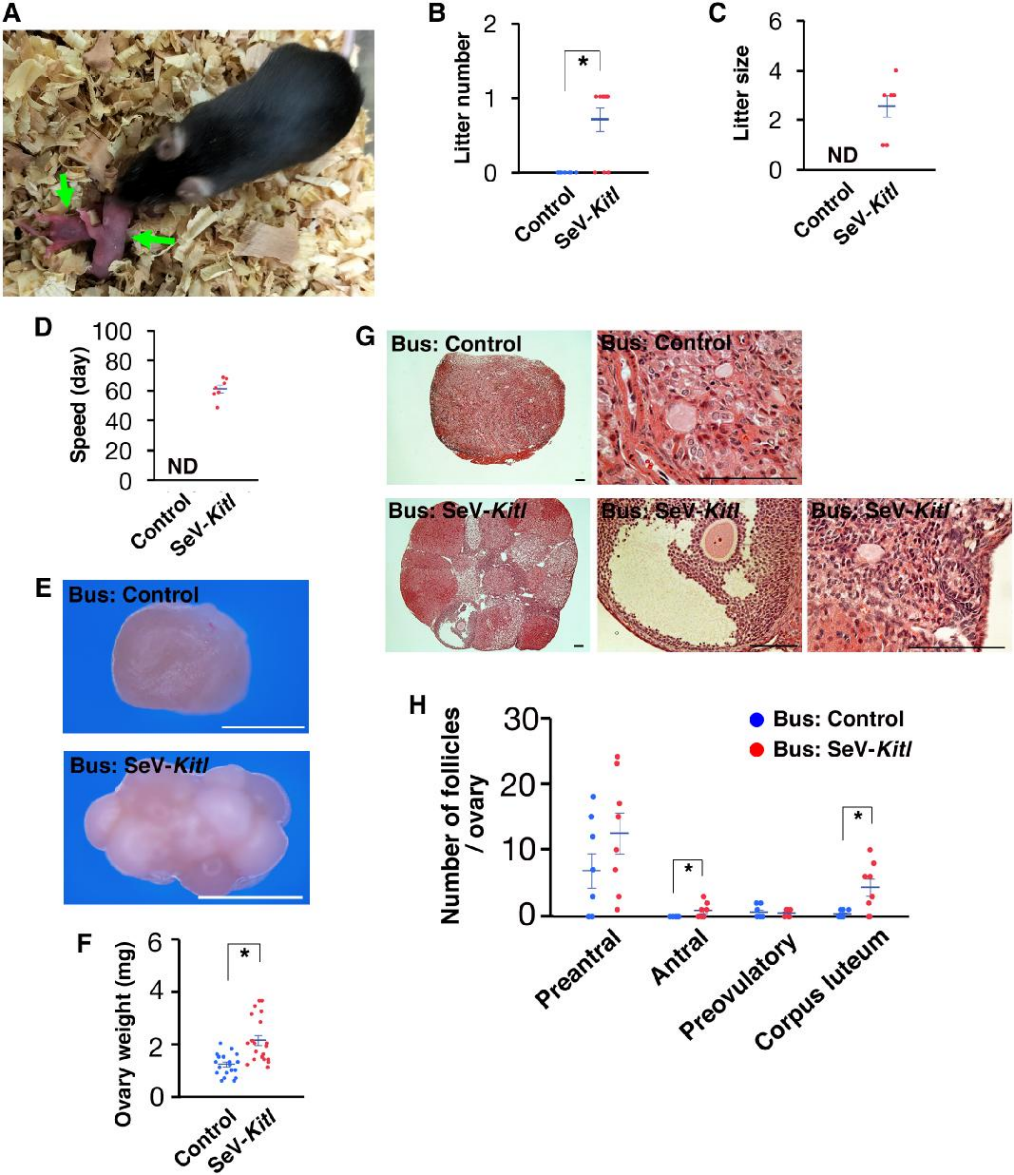
Fertility protection of wild-type mice against busulfan by SeV-*Kitl* injection. A) Offspring (arrows) born after mating with wild-type B6 males. B) Litter number (*n* = 10). Data are represented as mean ± SEM. C) Number of offspring per litter (*n* = 7). Data are represented as mean ± SEM. D) Speed of offspring production (*n* = 7). Data are represented as mean ± SEM. E, F) Appearance (E) and weight (F) of the ovaries 3 months after SeV-*Kitl* injection (*n* = 20). Data are represented as mean ± SEM. G) Histological analysis of follicle development. H) Analysis of follicle development (*n* = 8). Data are represented as mean ± SEM. Bar = 1 mm (E), 100 μm (G). Stain: hematoxylin and eosin (G).

While none of the control mice receiving SeV-*Egfp* sired offspring, 70% of mice that received SeV-*Kitl* became fertile (Fig. 5A and B), and sired one litter during the 3-month period. The size of the litters ranged from 1 to 4, with an average of 2.6 (Fig. 5C). Offspring were born as early as 48 days after busulfan injection (Fig. 5D).

These mice were sacrificed 3 months after busulfan injection for histological analysis. The size of the ovaries that received SeV-*Kitl* was significantly larger than those that received SeV-*Egfp* injection (Fig. 5E and F). Histological analyses also showed that ovaries that received SeV-*Kitl* developed numerous developing ovarian follicles (Fig. 5G). The number of antral follicles was significantly increased in ovaries that received SeV-*Kitl* injection (Fig. 5H). We also observed a large number of corpus luteum in these ovaries, reflecting successful ovulation. These results suggested that SeVs are useful for the protection of fertility against cancer treatment.

### Analysis of offspring

**Fig. 6.**
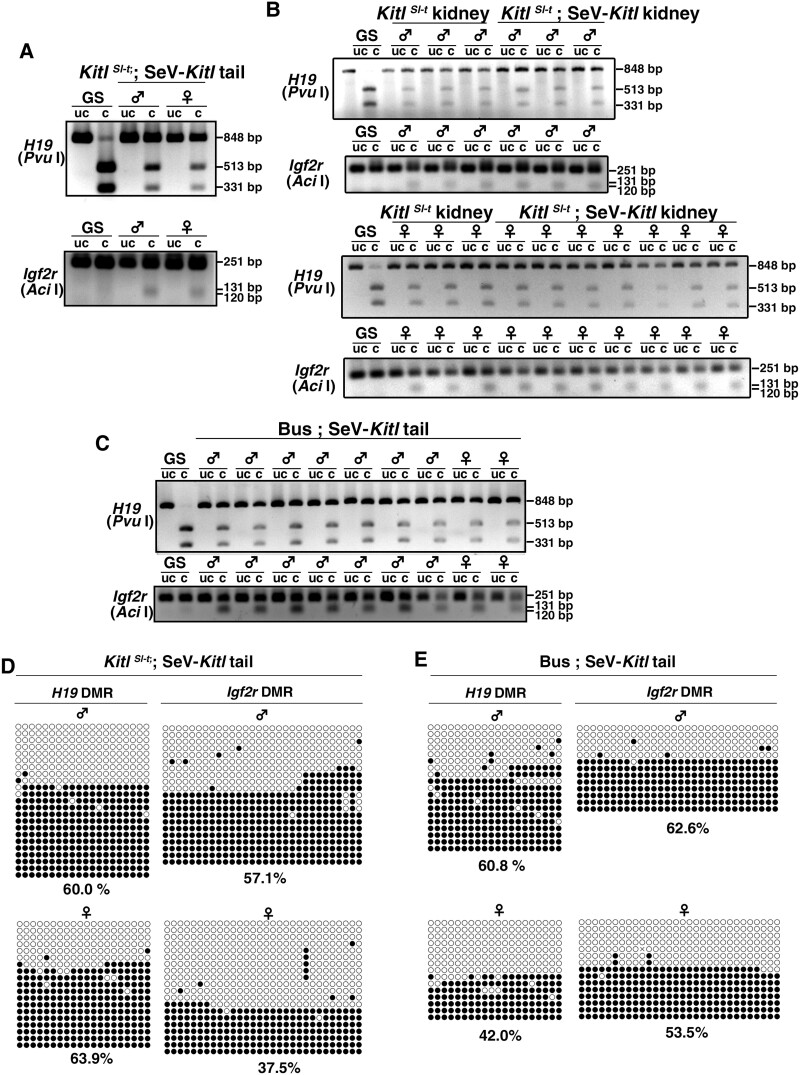
Analysis of genomic imprinting. A, B) COBRA of tail (A) or kidney (B) DNA from offspring born after SeV-*Kitl* injection into *Kitl^Sl-t^/Kitl^Sl-t^* mice. C) COBRA of tail DNA from offspring born after SeV-*Kitl* injection into busulfan-treated mice. D, E) Bisulfite sequencing of tail DNA born after SeV-*Kitl* injection into *Kitl^Sl-t^/Kitl^Sl-t^* or busulfan-treated mice. Germline stem (GS) cells, cultured spermatogonial stem cells.

As oocytes undergo gynogenetic genomic imprinting patterns during oogenesis (41), there was a possibility that ectopic expression of SeV-*Kitl* might lead to abnormal genomic imprinting patterns. To test this possibility, we collected tail and kidney DNA from *Kitl^Sl-t^/Kitl^Sl-t^* mice-derived offspring and performed combined bisulfite restriction analysis (COBRA). Analyses of differentially methylated region (DMR) of *H19* and *Igf2r* imprinted genes revealed DNA methylation patterns typical of somatic cells (Fig. 6A and B). Similar results were obtained for tail DNA from busulfan-treated mice (Fig. 6C). Normal genomic imprinting patterns of tail DNA were confirmed by bisulfite sequencing of DMRs of *H19* and *Igf2r* genes (Fig. 6D and E). These results suggested the maintenance of normal genomic imprinting patterns in the offspring.

Demonstrating that genomic imprinting is maintained in the tails or kidneys of offspring provides minimal assurance that the offspring are normal because the number of CpGs assayed is a very small subset of the methylome. To determine the long-term effects of SeV-mediated therapy, we performed metabolic analysis of the blood samples from F1 offspring. Given that ovulation induction and embryo manipulation can cause metabolic abnormalities in both mouse and human offspring (42, 43), we hypothesized that SeV-*Kitl* overexpression in *Kitl^Sl-t^/Kitl^Sl-t^* mutant mice might induce similar abnormalities because they had never ovulated since birth.

Serum biochemical parameters were measured at 8 months after birth. Of the 18 serum biochemical values examined, glucose and high-density lipoprotein were significantly higher in the offspring born after SeV treatment (Figure S7). Despite such abnormalities, the offspring were fertile and sired F2 and F3 generations by natural mating (Figure S8A). Their ovaries were small as their original and contained only primordial follicles *Kitl^Sl-t^/Kitl^Sl-t^* ovaries (Figure S8B and C).

## Discussion

While the feasibility of rescuing congenitally infertile mice using AAVs, AVs, and LVs has been demonstrated previously (13–18), these virus vectors may integrate into the genome of the oocyte, posing a major concern for gene therapy in infertility. Despite the success in mice, addressing this risk of genomic integration remains challenging, given compelling data on viral integration in somatic cells of various animal species (19, 20). Therefore, developing new strategies for infertility treatment that carry no risk of genotoxicity remains a prerequisite.

One unexpected finding in this study was the sustained expression of SeVs. Typically, achieving long-term expression in immunocompetent animals with RNA virus infection is challenging due to strong immune responses, making stable and persistent gene expression difficult or impossible in such settings (24). Most RNA virus vectors are not considered suitable for stable and persistent gene expression due to their potent cytotoxity triggered by antivirus responses, including the induction of *Ifnb* in host cells (25). However, our investigation of gene expression related to innate immunity revealed no apparent inflammatory reactions to SeV infection. Furthermore, the absence of hemagglutination and hemolysis in ovaries, where only a small amount of SeVs was directly introduced into the stroma, suggests that ovaries provide a hospitable microenvironment for gene therapy, overcoming the limitations observed in other tissues (26, 27).

Another potentially critical factor contributing to the successful restoration of fertility was the defective BFB in *Kitl^Sl-t^/Kitl^Sl-t^* mice. The BFB limits the diffusion of proteins larger than ∼20 nm in size (32). Previous studies demonstrated the successful infection of ovarian follicles using AAVs, with a diameter of ∼25 nm (44). This success was attributed to the unique ability of AAVs to undergo transcytosis (15). However, the SeV diameter is ∼230 nm (33), far exceeding the BFB's size and that of endocytic vesicles. Therefore, we initially thought that SeV infection might not occur at all. However, our results using FITC-dextran provided an impetus for us to attempt SeV infection in *Kitl^Sl-t^/Kitl^Sl-t^* mice because we observed stronger staining. Although it is possible that immunostaining may not exhibit high sensitivity for detection, the heightened staining implies a potential impairment of the BFB in *Kitl^Sl-t^/Kitl^Sl-t^* mice. Despite the description of the BFB in 1958 (45), its nature has not been extensively investigated. Our findings raise the possibility that the BFB may be impaired in other types of congenital infertility, warranting further studies to characterize the nature of the BFB in various disease models.

Our hormonal response analysis indicated minimal changes after SeV infection. In normal mice, ovulation is triggered by the surge of LH from the pituitary gland (46). Given that *Kitl^Sl-t^/Kitl^Sl-t^* ovaries have never been exposed to normal HPG axis regulation after birth, it was likely that the HPG axis in these mice are still immature or abnormal. Indeed, no significant changes were observed in LH, FSH, and estrogen levels. Although most offspring were born around 60 days after injection, with ovulation likely occurring ∼40 days, these hormone levels remained stable throughout the experimental period. This suggests that ovulation likely occurred without increase in LH levels in the current model. While the reason for this phenomenon is not currently known, it is possible that hormonal response might not have been matured because they were congenitally infertile. In addition, oogenesis was initiated by *Kitl* overexpression in ovaries in the current model. This is in contrast to healthy wild-type animals, where the hormonal response is initiated by an increase in LH levels from the pituitary gland. Although physiological analysis of the HPG axis is generally performed using wild-type animals, our experimental system may offer a unique model to study the development and flexibility of the HPG axis during oogenesis and ovulation.

The most significant advantage of the current approach is the restoration of fertility through natural mating. IVF and ICSI are two widely used techniques for human infertility treatment (47, 48). Particularly, ICSI is valuable as it allows offspring production from a small number of sperm. Previous success in gene therapy of male infertile models were all based on IVF or ICSI because only a limited number of sperm could be recovered after gene transduction (13, 14, 17, 18). However, these techniques require mature gametes, limiting their application to a select number of patients. Moreover, recent findings with ICSI in mice showed transgenerational abnormalities (11). Because of the long reproductive cycles in humans, it is currently impossible to evaluate the clinical implication of ICSI. In contrast, our successful recovery of infertility through natural fertility from mice with primordial follicles is encouraging as this technique may be applicable to ovarian defects with immature oocytes.

Our attempts to apply SeVs for busulfan-induced infertility showed promising results for fertility protection against cancer treatment. With an increasing number of cancer survivors, fertility protection has become an emerging problem. The incidence of cancer in 15–39-year-old adolescents and young adults is 52.3 cases per 100,000 (7). Various approaches, including administration of GnRH analogs and cryopreservation of oocytes/ovarian tissues, have been developed. In particular, ovarian tissue cryopreservation is the only currently available procedure to prepubertal girls. However, these techniques have limitations, with the efficacy of GnRH analog still under debate and transplantation of ovarian grafts being limited by extensive follicle loss (estimated to be approximately two-thirds of the follicles) occurring through ischemic reperfusion injury following transplantation (49). Reintroduction of malignant cells may also occur with this technique. Our SeV-mediated fertility protection offers a new possibility for in vivo protection of oocytes, broadening the application of SeV-based infertility treatment. Because the damage to oocytes and the health of patients would be relatively mild compared with previously reported procedures, further testing of this approach with other animals or chemicals is warranted for future clinical application.

An important observation from the present study was the lack of apparent immune response in SeV-infected ovaries. Although it was previously assumed that ovaries are among the immune-privileged organs, subsequent studies revealed that allografts transplanted in ovaries are rejected (50), suggesting that ovaries and testes have different immune environments. It is known that SeV infection activates innate immunity. Because NOG mice, which lack an innate immune response, can accept the SeV virus (24), it was considered that innate immunity activation is responsible for rejection of SeVs. It has been reported that SeV induces the production of interferon by boosting the TLR3, TLR7, and TLR8 signaling pathways, leading to antiviral responses (51). However, granulosa cells express TLR4, TLR8, and TLR9 (52). Although we currently do not know which of these receptors is primarily responsible for antiviral response against SeVs, innate immunity might be attenuated in the absence of these receptors or their downstream molecules, allowing persistent infection. Further analysis of the immune response is required to test this hypothesis.

Although our analyses of the offspring showed normal genomic imprinting patterns in the F1 offspring, biochemical analysis of the peripheral blood showed significantly increased levels of glucose and HDL. These results suggest that offspring born from SeV therapy are not completely normal. However, abnormalities in biochemical characteristics are often found in offspring produced by IVF and nuclear cloning (42, 53). Long-lasting effects of IVF observed in mouse models include increased fasting glucose, impaired glucose tolerance, insulin resistance, and higher blood pressure. Similar abnormalities are also reported for human offspring (43). These abnormalities are considered to be caused by abnormal DNA methylation, which is induced by ovulation and in vitro culture. On the other hand, because fertilization occurred in vivo, abnormalities might have been caused by SeV-induced forced ovulation. However, mouse studies did not show abnormalities in glucose metabolism by ovulation stimulation (54). Although more extensive analyses using a larger sample size are needed to confirm and elucidate the mechanism underlying our findings, caution is necessary for clinical application, and monitoring long-term health problems in the offspring is a prerequisite. In particular, the impact on the lifespan should be investigated, given that IVF-derived offspring are short-lived depending on the composition of the diet (55).

Because establishing a causal relationship for congenital infertility is not always easy, iatrogenic infertility may be a good candidate to test the effectiveness of SeVs for clinical application because our results suggest that KITL may also attenuate human oocyte apoptosis. Although oocytes or ovarian cryopreservation are currently used, they require IVF or ICSI, whose risks remain unknown (11). The use of SeVs eliminates the risk of germline integration, a major hurdle in infertility gene therapy. As SeVs infection is relatively localized by binding to sialic acid (56), it provides an effective approach to avoid undesired systemic delivery into other tissues (33), which is another problem for gene therapy vectors. Although the high immunogenicity of SeVs has impeded their application, the unique immunomodulatory function of the ovaries may render them a suitable candidate for clinical application. However, they may develop unexpected side effects in the long-term, which needs to be examined by extensive animal experiments. Although anatomical and immunological differences in humans may pose challenges, our success in mice could potentially provide an alternative approach for human infertility treatent.

## Materials and methods

### Animals and virus injection

Our study examined female mice because the disease modeled is only relevant in females. We utilized 4- to 5-week-old B6 mice unless otherwise indicated. In some experiments, 4- to 8-week-old B6.Cg- Kitl/Rbrc mice with a mixed B6, DBA/2 background were employed (RIKEN BRC, Ibaraki, Japan). We also used 4- to 8-week-old W mice (Japan SLC, Shizuoka, Japan) and *Lhcgr* KO mice (gift from Dr Rao, C. V.; Florida International University) (37). All animal experiments were approved by The Institutional Animal Care and Use Committee of Kyoto University. Animals were maintained under conditions of ad libitum access to water and food with constant light–dark cycles. SeVs were obtained from ID Pharma (Egfp-SeV/TSdeltaF and mKitl-SeV/TSdeltaF; Tokyo, Japan).

Ovarian injections were carried out, as described previously (15). In brief, 2 ventral–lateral flank incisions were made ∼2-mm caudal to the last rib. By gently securing a fat pad around the oviduct and ovarian bursa, a glass needle was inserted under the tunica albuginea of the ovary. Approximately 2-μl of FITC-dextran (50 mg/ml; Sigma, St. Louis, MO, USA) or virus particles were microinjected into the ovarian stroma. For dextran injections, the ovaries were recovered 30 min after injection. For production of offspring, males were added at least 2 weeks after microinjection. Where indicated, busulfan was administered intraperitoneally (22 mg/kg).

### Analysis of DNA integration

To assess DNA integration of SeVs, tail DNA of the offspring was collected. PCR analysis was conducted using *Egfp*-specific primers (Table S1).

### Immunostaining

Immunostaining was performed, as previously described (57). Ovary samples were fixed in 4% paraformaldehyde for 2 h at 4°C, embedded in Tissue-Tek OCT compound (Sakura Finetek, Tokyo, Japan), and subjected to cryosectioning. To block nonspecific antibodies, sections were treated with 3% bovine serum albumin (BSA) and 10% donkey serum in phosphate-buffered saline (PBS) supplemented with 0.1% Tween 20 (PBST) for 1 h at room temperature. The sections were then incubated with the indicated primary and secondary antibodies with 0.5% BSA in PBST overnight and for 1 h, respectively. The sections were washed with PBST. The antibodies used in this analysis are listed in Table S2. Hoechst 33342 (Sigma) was used for counterstaining.

### Real-time PCR analysis

Real-time PCR was performed, as described previously (57). Total RNA was isolated using TRIzol reagent (Invitrogen, Carlsbad, CA, USA). First-strand cDNA was generated using a Verso cDNA synthesis kit for RT-PCR (Thermo Fisher Scientific, Waltham, MA, USA). For real-time PCR, the StepOnePlus real-time PCR system (Applied Biosystems, Warrington, United Kingdom) and *Power* SYBR Green PCR Master Mix (Applied Biosystems) were used according to the manufacturers' protocols. Transcript levels were normalized to those of *Hprt*. The PCR conditions were as follows: 95°C for 10 min, followed by 40 cycles at 95°C for 15 s and 60°C for 1 min. Each PCR was performed at least three times. The primers used are listed in Table S1.

### Serum analysis

Biochemical parameters were measured using an automatic biochemical analyzer (JCA-BM6070; JEO0L Ltd, Tokyo, Japan).

### COBRA

COBRA was performed, as described previously (15). Genomic DNA was treated with sodium bisulfite, which deaminates unmethylated cytosines to uracils but does not affect 5-methylated cytosines. The DNA was used as a template to amplify DMRs using specific primers. The PCR product was digested with the indicated restriction enzymes, which recognize DNA sequences containing CpG in the original unconverted DNA. The primers used are listed in Table S1.

### Bisulfite sequencing

Bisulfite sequencing was performed as described previously (15). Bisulfite-treated DNA was amplified by PCR using specific primer sets. DNA methylation analyses were performed using the Quantification Tool for Methylation Analysis (QUMA) (http://quma.cdb.riken.jp/top/quma_main_j.html). The primers used are listed in Table S1.

### Statistical analysis

Significant differences between means for single comparisons were determined by Student's t tests. All data were analyzed by Microsoft Excel. All the results are expressed as mean ± SEM. A value of *P* < 0.05 was considered to be statistically significant.

## Supplementary Material

pgae375_Supplementary_Data

## Data Availability

All data associated with this study are present in the paper or the Supplementary material.
