# Supplementary material for: Sendai virus-mediated RNA delivery restores fertility to congenital and chemotherapy-induced infertile female mice
Source: PNAS Nexus. 2024 Sep 3;3(9):pgae375. doi: 10.1093/pnasnexus/pgae375 (PMC11388103; doi:10.1093/pnasnexus/pgae375)
Supplement: pgae375_Supplementary_Data [file pgae375_supplementary_data.pdf]

Supplementary Information for

Sendai virus-mediated RNA delivery restores fertility to congential and chemotherapy-induced infertile female mice

Mito Kanatsu-Shinohara, Hiroko Morimoto, Tianjiao, Liu, Masaru Tamura, Takashi Shinohara

Takashi Shinohara
Email: tshinoha@virus.kyoto-u.ac.jp

This PDF file includes:

Figures S1 to S8
Tables S1 to S2

Figure S1

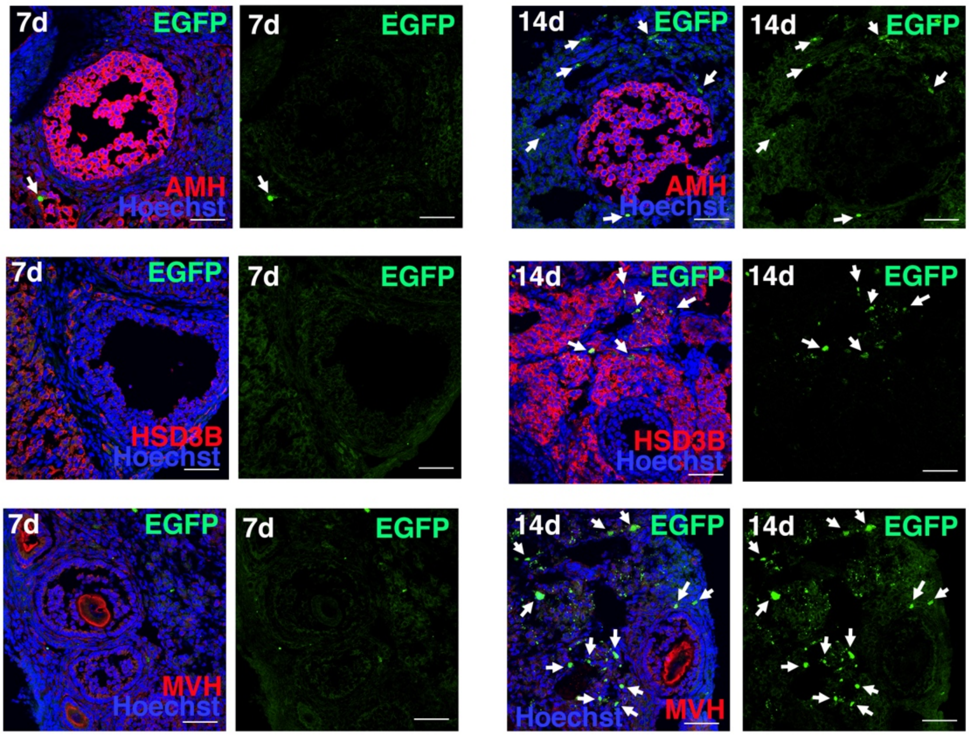

Fig. S1. EGFP expression in infected ovaries. Immunostaining of ovaries that received SeV-*Egfp* injection. Bar = 50 μm. Stain: Hoechst33342.

Figure S2

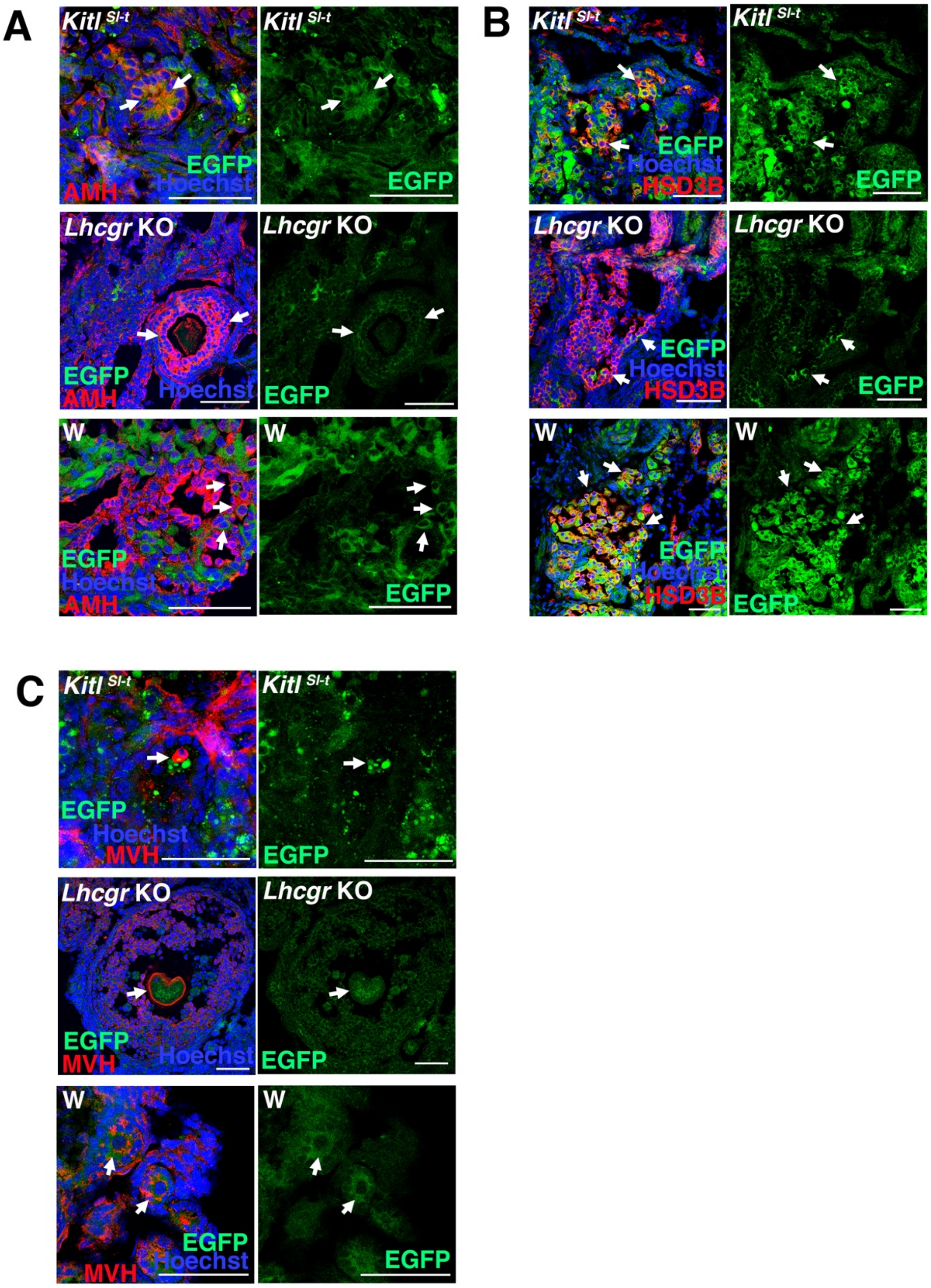

Fig. S2. SeV injection into mutant mice. Immunostaining of ovaries that received SeV-*Egfp* injection. Ovaries from *Kitl*$^{Sl\text{-}t}$/*Kitl*$^{Sl\text{-}t}$, *Lhcgr* KO, and W mice were stained with antibodies against AMH (granulosa cell marker; A), HSD3B (theca cell marker; B), and MVH (oocyte marker; C), respectively. Arrows indicate cells that express both EGFP and target antigens. Bar = 50 μm. Stain: Hoechst33342.

Figure S3

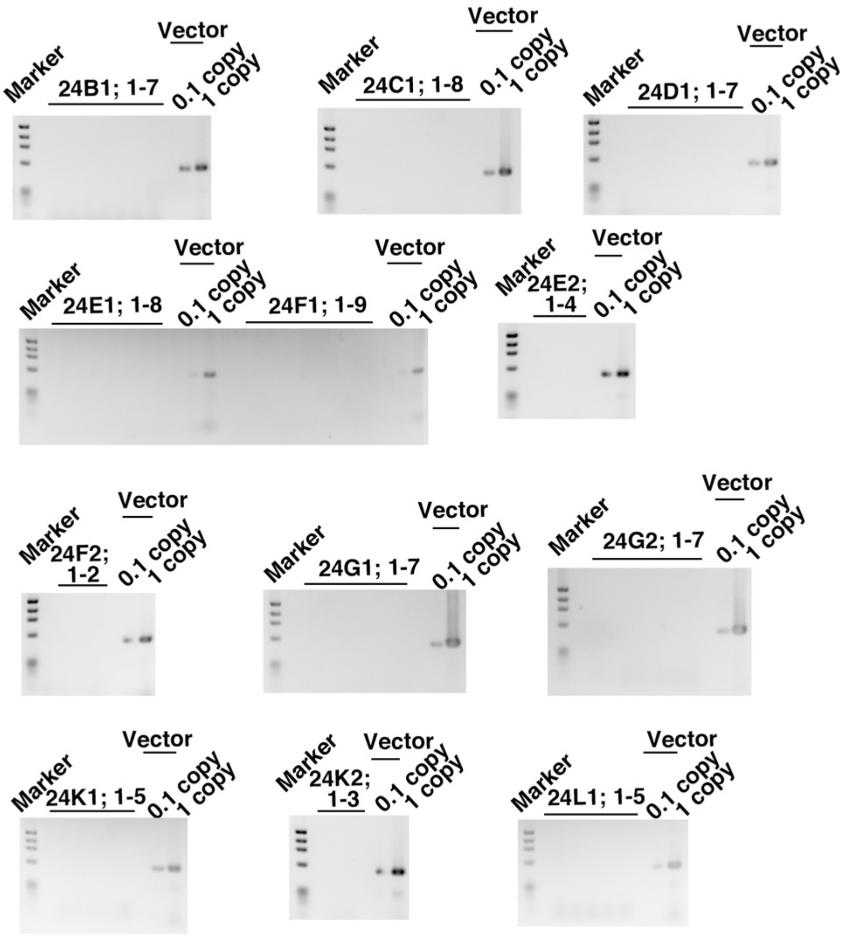

Fig. S3. PCR analysis of SeV integration. *Egfp*-specific primers were used to amplify *Egfp* transgene in tail DNA of offspring.

Figure S4

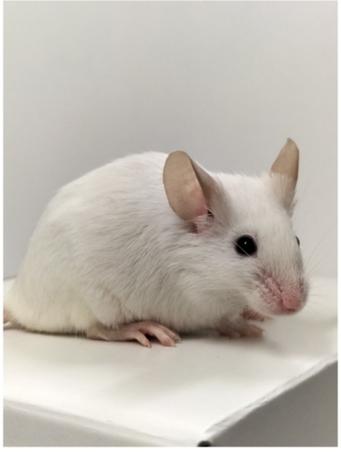

Fig. S4. Appearance of mature F1 offspring sired by natural mating between *Kitl*$^{Sl-t}$/*Kitl*$^{Sl-t}$ male and female. White coat color indicates the loss of melanocytes in this strain.

Figure S5

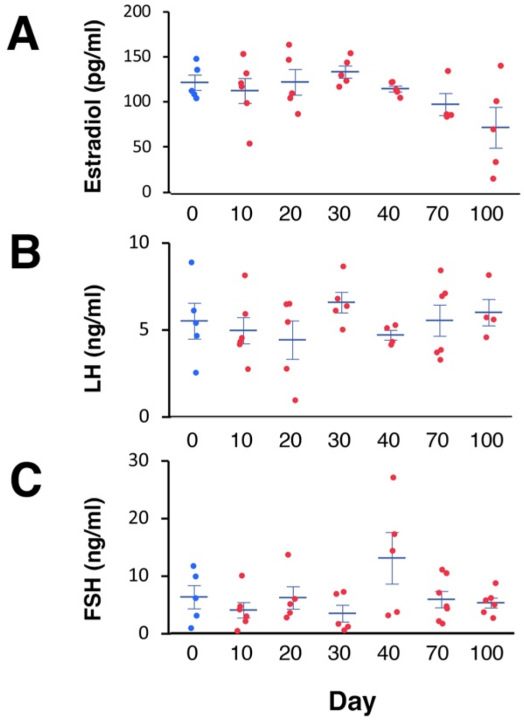

Fig. S5. Hormone levels in peripheral blood. (A) Estradiol (n = 4-6). (B) LH (n = 4-6). (C) FSH (n = 5-7).

Figure S6

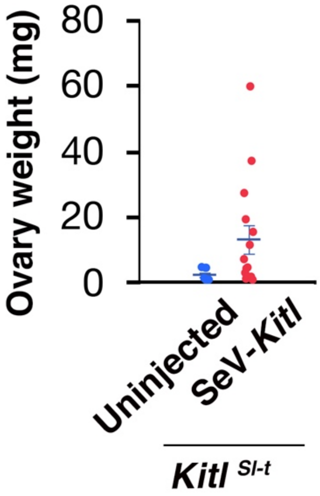

Fig. S6. Size of the ovary 6 months after SeV-*Kitl* injection (n = 8 for control, n = 15 for SeV-*Kitl*).

Figure S7

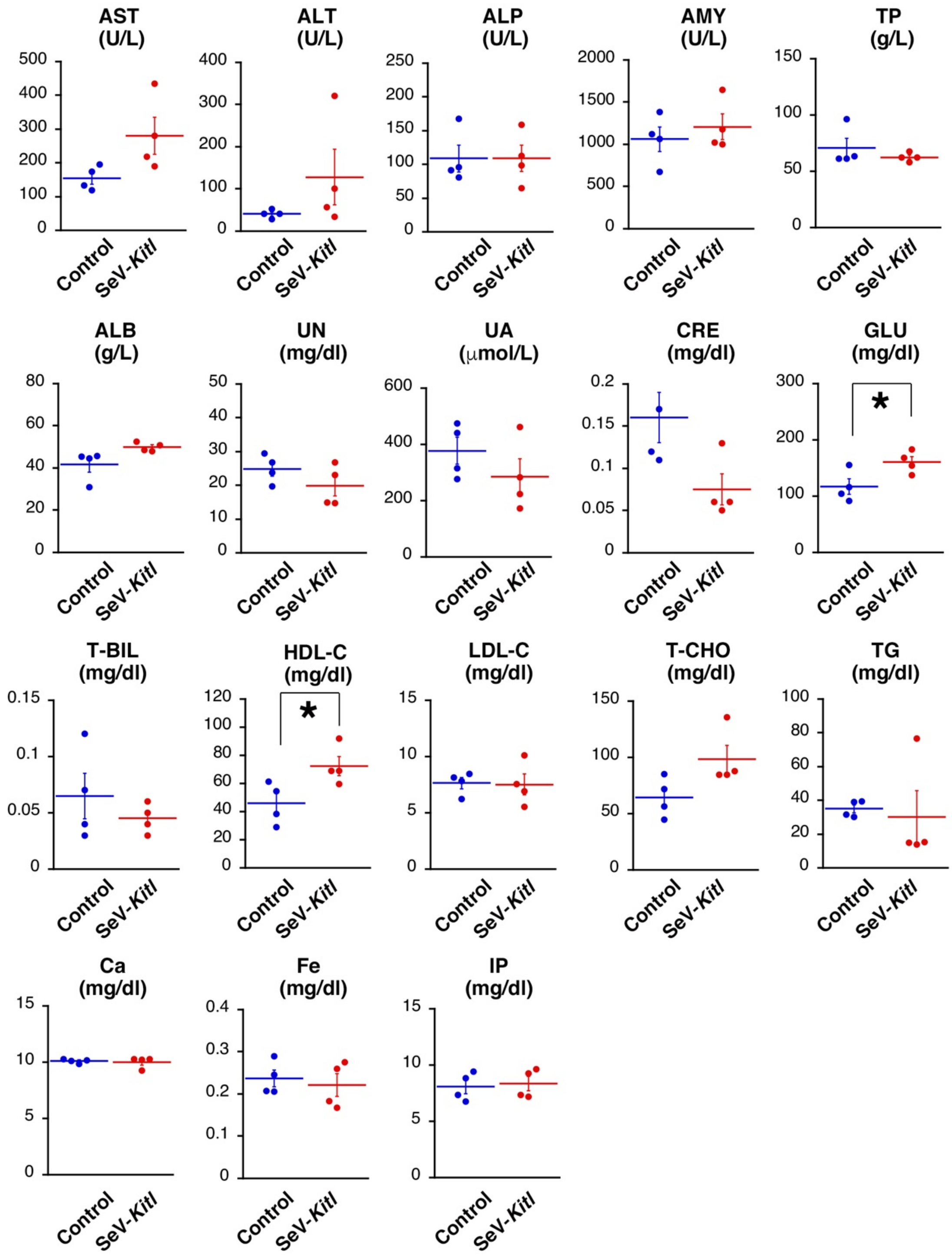

Fig. S7. Serum biochemical analysis (n = 4).

Figure S8

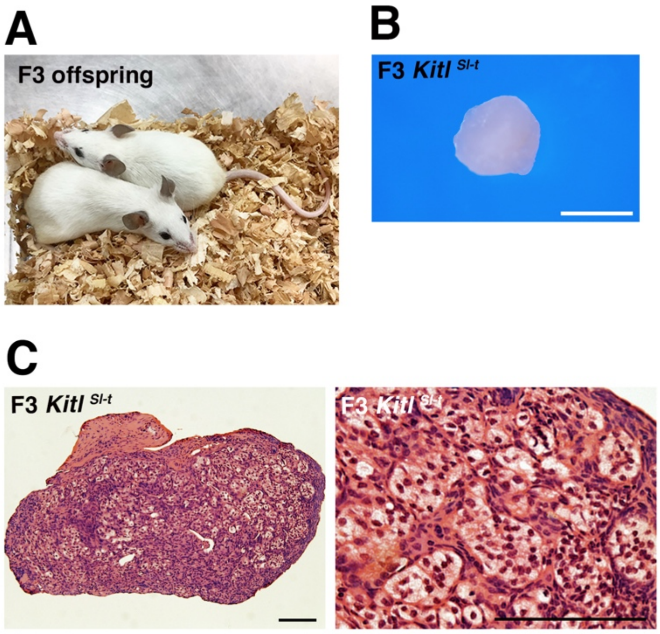

Fig. S8. Germline transmission of mutant phenotype. (A) Appearance of mature F3 offspring sired by natural mating between heterozygous F2 offspring. White coat color indicates the loss of melanocytes in this strain. (B) Macroscopic appearance of F3 ovary. (C) Histological appearance. Bar = 1 mm (B), 100 μm (C).

Table S1. PCR primers used in the present study

**Integration of Sendai virus**		
**Forward**		**Reverse**
ATGGCCGGGTTGTTGAGCAC		GTCTTGTCTGAACGCCTCTAAC
**RT-PCR**	**Forward**	**Reverse**
Hprt	GCTGGTGAAAAGGACCTCT	CACAGGACTAGAACACCTGC
Kitl	GATCTGCGGGAATCCTGTGA	ACTAGGCAAAACATCCATCC
**qPCR**	**Forward**	**Reverse**
Hprt	GCTGGTGAAAAGGACCTCT	CACAGGACTAGAACACCTGC
Kitl	GATCTGCGGGAATCCTGTGA	ACTAGGCAAAACATCCATCC
Il6	CCAGAGTCCTTCAGAGAGAT	GAATTGGATGGTCTTGGTCC
Tnfa	AGTTCTATGGCCCAGACCCT	ACAAGGTACAACCCATCGGC
Ifna4	CTACTGGTCAGCCTGTTCTC	GTTGGTTATCCACCTTCTCC
Ifnb1	GAACAACAGGTGGATCCTCC	GGCAGTGTAACTCTTCTGCA
Rig1	TCAGCTACATGAGTTCCTGG	TGCATGGTACAGGGCATCCA
Ifih1	TGTCTTGGACACTTGCTTCG	CCACCTGTTAGTTCTTGGAA
Tlr3	GAACCTCCAAGAACTGCTCT	TAGCCAGAGAGAGATTCTGG
**COBRA**	**Forward**	**Reverse**
H19	GGAATATTTGTGTTTTTGGAGGG	TTAAACCCCAACCTCTACTTTTATAAC
Igf2r	TTAGTGGGGTATTTTTATTTGTATGG	AAATATCCTAAAAATACAAACTACACAA
**Bisulfite-seq**	**Forward**	**Reverse**
H19 1 st	TTTGGGTAGTTTTTTTAGTT	TCCTAATCTCTAATCTCAAC
H19 2 nd	TTTGGGTAGTTTTTTTAGTT	AACCCCAACCTCTACTTTTA
Igf2r 1 st	TAGAGGATTTTAGTATAATTTTAA	CACTTTTAAACTTACCTCTCTTAC
Igf2r 2 nd	GAGGTTAAGGGTGAAAAGTTGTAT	CACTTTTAAACTTACCTCTCTTAC

Table S2. Antibodies used in the present study

**Immunofluorescence**		
**Antigen**	**Name**	**Company**
AMH	Goat anti-human MIS (AMH)	Santa Cruz Biotechnology (sc-6886)
HSD3B	Rabbit anti-mouse HSD3B	Trans Genic Inc. (KO607)
MVH	Rabbit anti-human DDX4/MVH	Abcam (ab13840)
CD4	Rat anti-mouse CD4	eBioscience (clone GK1.5)
CD8	Rat anti-mouse CD8	Abcam (ab22378)
MKI67	Alexa Fluor 647-conjugated mouse anti-MKI67	BD Biosciences (5458615)
**Secondary reagents**		
Alexa Fluor 555-conjugated donkey anti-rabbit IgG		Invitrogen (A31572)
Alexa Fluor 555-conjugated donkey anti-goat IgG		Invitrogen (A21432)
Dylight 550-conjugated donkey anti-rat IgG		Invitrogen (SA5-10027)
Alexa Fluor 647-conjugated donkey anti-rabbit IgG		Invitrogen (A31573)
Alexa Fluor 647-conjugated donkey anti-goat IgG		Invitrogen (A21447)
In Situ Cell Death Detection Kit, TMR red		Roche 12156792910
